# Method Based on Ultrafiltration to Obtain a Plasma Rich in Platelet and Plasma Growth Factors

**DOI:** 10.3390/jcm12185941

**Published:** 2023-09-13

**Authors:** Jon Mercader Ruiz, Maider Beitia, Diego Delgado, Pello Sánchez, Jorge Guadilla, Cristina Pérez de Arrilucea, Fernando Benito-Lopez, Lourdes Basabe-Desmonts, Mikel Sánchez

**Affiliations:** 1 Arthroscopic Surgery Unit, Hospital Vithas Vitoria, 01008 Vitoria-Gasteiz, Spain; jon.mercader@ucatrauma.com (J.M.R.); pello.sanchez@ucatrauma.com (P.S.); jorge.guadilla@ucatrauma.com (J.G.); cristina.perez@ucatrauma.com (C.P.d.A.); 2 Microfluidics Cluster UPV/EHU, BIOMICs Microfluidics Group, Lascaray Research Center, University of the Basque Country UPV/EHU, 01006 Vitoria-Gasteiz, Spain; 3 Advanced Biological Therapy Unit, Hospital Vithas Vitoria, 01008 Vitoria-Gasteiz, Spain; maider.beitia@ucatrauma.com (M.B.); diego.delgado@ucatrauma.com (D.D.); 4 Microfluidics Cluster UPV/EHU, Analytical Microsystems & Materials for Lab-on-a-Chip (AMMa-LOAC) Group, Analytical Chemistry Department, University of the Basque Country UPV/EHU, 48940 Leioa, Spain; fernando.benito@ehu.eus; 5 Basque Foundation of Science, IKERBASQUE, 48009 Bilbao, Spain

**Keywords:** platelet-rich plasma, hydrogel, water filtration, platelets, biomolecules, growth factors

## Abstract

Platelet-Rich Plasma (PRP) is an autologous biological product which, due to its regenerative capacity, is currently used in different fields of medicine. This biological treatment has proven to be effective in numerous research studies due to its high content of growth factors released by platelets. However, the current systems used to obtain PRP do not enrich the growth factors and cytokines outside platelets. Considering this, the present work aims to develop a new technique by which all the biomolecules present in plasma are enriched. Thus, a new method based on ultrafiltration has been developed for the obtaining of the novel PRP. By this method, ultrafiltration of the plasma water is carried out using a 3KDa filtering unit. The results showed that the technique was able to concentrate extraplatelet factors, such as IGF-1 and HGF, in contrast with conventional plasmas. Thus, the cultured cells responded with increased viability to this new PRP. These results could provide a new approach to the treatment of injuries requiring regenerative medicine, potentially improving the outcomes of the conventional PRPs.

## 1. Introduction

Platelet-Rich Plasma (PRP) is a biological hemoderivative product obtained from blood in which platelets are present in a higher concentration than in basal levels. Its restorative potential is mainly based on platelets, which are anucleate blood elements with a diameter of approximately 3 µm and derived from the hematopoietic line via the megakaryocyte, having a key role in hemostasis and thrombosis [1].

Platelets release an arsenal of potential regenerative and mitogenic substances that are involved in wound healing and play a key role in tissue regeneration [2]. These released substances mediate many cellular functions, including cell migration, differentiation, cellular cycle, apoptosis, metabolism, as well as proliferation [3,4,5]. However, some growth factors (GF), like the Insulin-like Growth Factor-1 (IGF-1) and the Hepatocyte Growth Factor (HGF), are outside of the platelets in plasma and promote wound healing, bone formation, myogenesis of striated muscle and keratinocyte migration, and have antifibrotic and anti-inflammatory properties. Besides molecules, structures such as macrovesicles and exosomes [6] also circulate within the plasma, being essential in processes related to cell communication and signaling [7]. Therefore, despite the importance of platelets and their derivates, other extraplatelet components must be carefully considered in the therapeutic potential of PRP. In a very recent study, it was found that higher values of IGF-1 are directly related to an increase in cell proliferation [8]. Combining that effect with the concentration of other molecules with the capacity to inhibit transcription and translation of pro-inflammatory genes and proteins, as HGF does [9,10], the PRP treatment could be improved. However, obtaining such a formula is not straightforward. At present, PRP preparation techniques involve several centrifugation steps to obtain a plasma fraction with a platelet concentrate [11]. There are few alternative systems to centrifugation to obtain PRP, and they are mainly focused on reducing sample handling and obtaining higher volumes of PRP in a reduced time. They make use of sedimentation, filtration, and even ultrasound technology to separate the different cellular components. The vast majority of these systems end up coupling filters to acquire a purer PRP focusing only on platelet concentration without taking into account other molecular components of the plasma [12,13,14,15,16,17].

Most of these devices aim to remove the red blood cells (RBC) and white blood cells (WBC) in the separation process before concentrating the platelets in plasma. For the efficient separation of PRP, these systems require a sample dilution step of the whole blood, favoring the separation of RBCs and WBCs to obtain at last a final product with a high platelet and GF concentration [12,13,15].

Among all the commercial kits for PRP obtaining, there is a system on the market capable of eliminating RBCs and WBCs, enriching platelets to obtain PRP and, in turn, concentrating the extraplatelet biomolecules of a molecular weight greater than 25 KDa but leaving other biomolecules below that limit which could be key to achieve that balance between platelet and extraplatelet GFs [18].

In this manuscript, we report a new PRP production method, based on the ultrafiltration of water from the plasma, for the obtaining of a novel PRP (nPRP) enriched in both platelet and extraplatelet biomolecules, including those with a molecular weight (MW) lower than 25 KDa.

## 2. Materials and Methods

### 2.1. Donors

Eight healthy donors were selected, ranging in age from 29 to 58 years old (4 female/4 male). Ethical approval was obtained from the Ethics Committee of UPV/EHU (2019-234, 13 May 2020), and written consent was obtained from the patients.

### 2.2. Standard Platelet-Rich Plasma (sPRP) Preparation

Whole blood was withdrawn into tubes of 9 mL and 3.5 mL, containing 3.8% (*w*/*v*) sodium citrate. Then, 3.5 mL tubes were used to measure platelet concentration at baseline levels, while the others were used to obtain the sPRP and the nPRP. Furthermore, 9 mL of blood was centrifuged at 580× *g* for 8 min at room temperature (RT). To obtain the sPRP fraction, the first 2 mL of plasma over the buffy coat was collected. For platelet activation, 10% CaCl_2_ (aq) (20 µL mL^−1^) was added to the plasma, which is the minimum dose used for clot formation [19].

### 2.3. Novel Platelet-Rich Plasma (nPRP) Preparation

**Figure 1:**
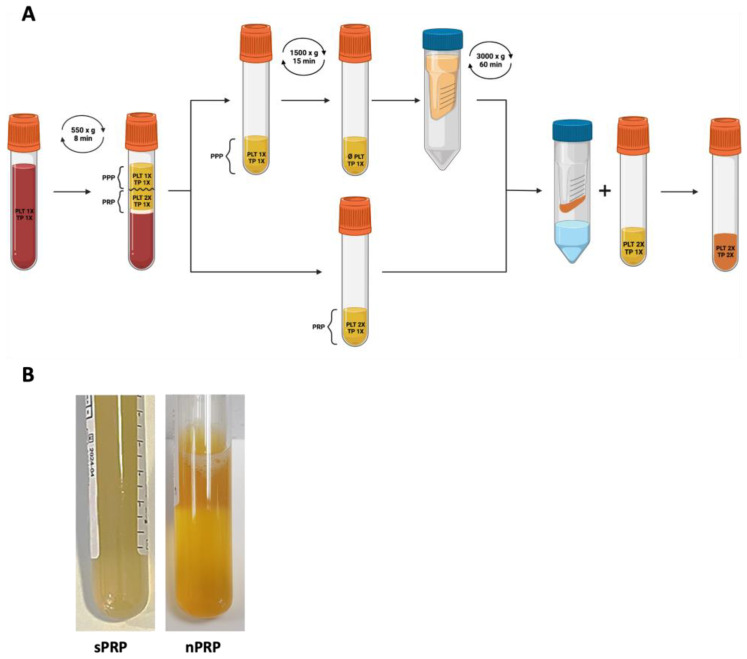
nPRP obtaining protocol. (**A**) The protocol to obtain the nPRP consists of first centrifugation where PRP and PPP are obtained. The PPP undergoes new centrifugation to precipitate the platelets from the plasma, and this new platelet-free plasma is passed through a 3 KDa filter by centrifugation. The remnant present in the filter is finally dissolved in the PRP obtained in the first step. PLT: expected platelet content; TP: expected total protein. (**B**) Clinical photographs of sPRP and nPRP formulations.

Whole blood was withdrawn into tubes of 9 mL and 3.5 mL, containing 3.8% (*w*/*v*) sodium citrate. Then, 3.5 mL tubes were used to measure platelet concentration at baseline levels, while the others were used to obtain the sPRP and the nPRP. The nPRP was obtained by centrifuging the 9 mL tubes of whole blood at 550× *g* for 8 min at RT as in the sPRP process. After the separation of the plasma fraction from the rest of the components, a gradient with an increasing platelet concentration (PLT) downwards was generated. PRP consisted of the first 2 mL plasma above the buffy coat. The remaining plasma fraction was a platelet-poor plasma (PPP) fraction. For these analyses, 6 mL of each fraction was collected and transferred to a new tube. Subsequently, the PPP was centrifuged at 1500× *g* for 15 min at RT to obtain plasma without platelets. Then, this fraction was passed through an Amicon 3 KDa ultrafiltration filter unit (Millipore, Burlington, MA, USA) and centrifuged at 3000× *g* for 60 min. The protein concentrate that remained in the filter was dissolved in the previously obtained PRP (6 mL) (Figure 1A). Finally, 10% CaCl_2_ (20 µL mL^−1^) was added to the plasma to trigger clot formation. Figure 1B shows clinical photographs of sPRP and nPRP formulation before platelet activation.

### 2.4. Haematology and Protein Parameters

Platelet, total protein, and albumin levels were measured in whole blood, sPRP, and nPRP samples from all the donors. Platelets were measured with a hematology analyzer (Sysmex XS-1000i; Kobe, Japan), whereas total protein and albumin levels were measured with a Cobas c 501 analyzer (Roche, Basel, Switzerland).

### 2.5. Ion Concentration Analysis

Ion concentration measurements were carried out for sPRP and nPRP plasma samples with the Cobas c 501 analyzer. Ca^2+^, Na^+^, Cl^−^, Mg^2+^, PO_4_^3−^, and K^+^ were the selected ions to be measured.

### 2.6. Platelet Activation Test

Measurement of P-selectin was used as a method to test the platelet activation. P-selectin, also called CD62P, is stored in α-granules of inactivated platelets. After platelet activation, the inner walls of these granules were exposed on the outside of the cells, presenting the CD62P protein [20]. For that purpose, CD41 and CD62P antibodies, which recognize platelet membrane constitutive glycoprotein GpIIb and translocated p-selectin, respectively, were used.

For each donor, 10 μL of plasma sample, 5 μL of anti-CD41-FITC, and 5 μL of anti-CD62P-PE (BD Biosciences, San Jose, CA, USA) were added in the test tubes. The remaining volume of up to 100 μL was completed with PBS. Finally, samples were incubated for 15 min at RT in the dark and then were fixed with 400 μL of freshly prepared 1.25% formaldehyde (PanReac AppliChem, Barcelona, Spain) in PBS (Gibco, Waltham, MA, USA). A Gallios flow cytometer (Beckman-Coulter, High Wycombe, UK) was used to analyze the events.

### 2.7. Quantification of GFs

Both platelet and plasmatic GFs present in sPRP and nPRP were analyzed. Among the wide variety of GFs, PDGF-AB, IGF-1, and HGF were analyzed. Plasma samples were activated by adding CaCl_2_ to allow for the release of GFs from platelets and quantify them in the serum. The concentration of those GFs was measured using a commercially available enzyme-linked immunosorbent assay kit (ELISA) (R&D Systems, Minneapolis, MN, USA).

### 2.8. Cell Culture

Normal human dermal fibroblast (NHDF) (Lonza, Basel, Switzerland) was kept in the incubator at 37 °C and 5% CO_2_ atmosphere. Cells were grown in fibroblast growth basal medium (FBM, Lonza, Basel, Switzerland) supplemented with insulin, human fibroblast GF, and gentamicin sulfate–amphotericin at 0.1% (*v*/*v*) each (Lonza, Basel, Switzerland), as recommended by the manufacturer.

### 2.9. Cell Viability Assays

For the evaluation of the biological activity of both sPRP and nPRP, NHDFs were incubated with FBM medium supplemented with FBS in 96-well white flat-bottom microplates (Greiner, Kremsmünster, Austria) at a density of 1  ×  10^3^ cells per well in 200 µL culture medium. After 24 h, cells were treated with 10% of either sPRP or nPRP and after 96 h, the viability was measured in triplicate by Real Time-Glo MT Cell Viability Assay (Promega, Fitchburg, MA, USA). This method is based on the reducing potential of metabolically active cells that catalyze the conversion of a synthetic substrate into a luminescent product, and the level of luminescence can be considered proportional to the number of viable cells present in the assay [21]. Luminescence reading was performed by a TECAN Infinite 200 PRO plate reader (TECAN, Zurich, Switzerland).

### 2.10. Statistical Analysis

The distribution of the samples was assessed by Shapiro–Wilk’s normality test. The different variables were determined by the mean and the standard deviation for parametric data and median and 95% confidence interval (CI) for non-parametric data. Comparisons were performed by ANOVA, Student’s *t*-test for parametric data, and Kruskal–Wallis and Mann–Whitney U test for non-parametric data. Data were considered statistically significant when *p* < 0.05. GraphPad Prism^®^ software version 9.5 (San Diego, CA, USA) was used for the statistical analysis.

## 3. Results

### 3.1. nPRP Characterization

**Figure 2:**
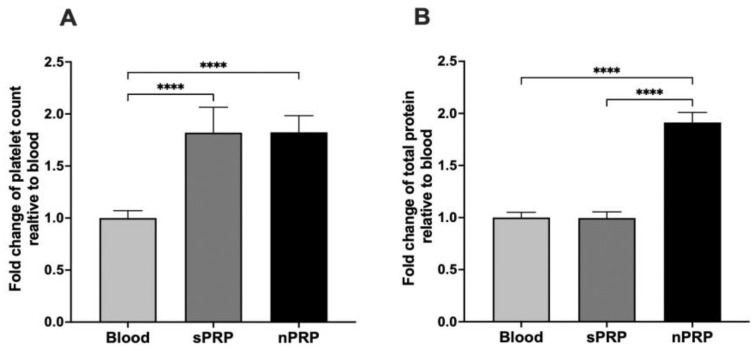
Platelet and total protein fold change in sPRP and nPRP relative to blood. (**A**) Platelet and (**B**) total protein fold change values are expressed as mean ± standard deviation of 8 donors. Statistical significance was calculated by one-way ANOVA (**** *p* < 0.0001).

The sPRP and nPRP showed a double number of platelets compared to blood (*p* < 0.0001 in both cases), and there were no differences between the two PRPs (Figure 2A). According to the classification and coding system made by Kon et al. [22], both plasmas have the classification code 13-00-11. The code is a sequence of six digits grouped in pairs indicating parameters of PRP composition relative to platelets, purity, and activation with the aim of unifying the way of classifying PRP for comparative purposes.

In terms of total protein concentration, there was no statistical significance between blood and sPRP, whereas nPRP total protein concentration was almost double, making these differences statistically significant (*p* < 0.0001 in both cases) (Figure 2B).

### 3.2. Determination of Ion Levels in Plasma Samples

**Figure 3:**
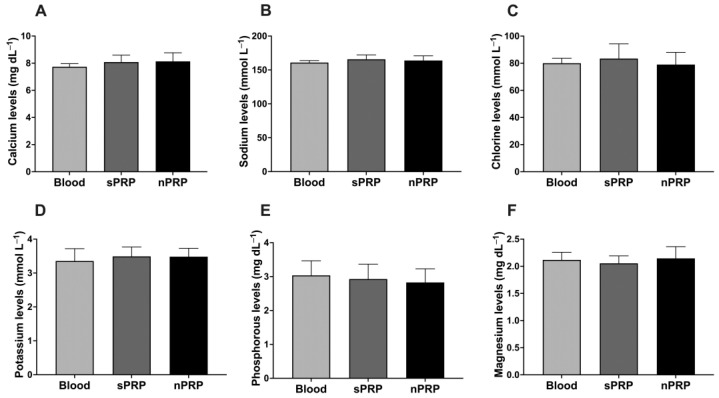
Ion levels in blood, sPRP, and nPRP. (**A**) Calcium, (**B**) sodium, (**C**) chlorine, (**D**) potassium, (**E**) phosphorous, and (**F**) magnesium levels are expressed as mean ± standard deviation of 8 donors. Statistical significance was calculated by one-way ANOVA.

Regarding the ion concentration in nPRP obtained after filtration, there was no change in their concentration with respect to basal levels. No statistical significance was achieved comparing the ion levels between nPRP and blood or sPRP (Figure 3A–F).

### 3.3. P-Selectin Expression Detection for Activated Platelet Detection

**Figure 4:**
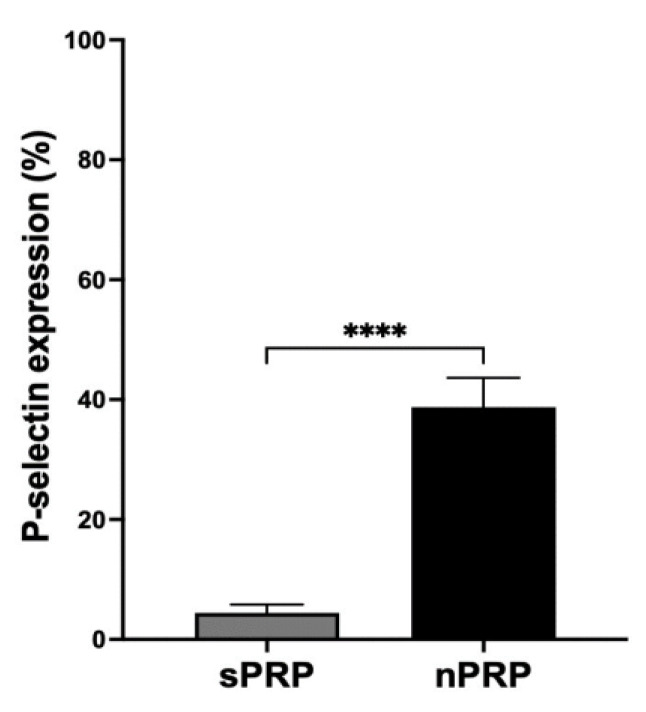
Platelet activation analysis by P-selectin expression measurement. P-selectin expression values are expressed as mean ± standard deviation of 8 donors. The statistical significance was calculated by *t*-test (**** *p* < 0.0001).

Platelet activation test showed that the expression of P-selectin is significantly increased in nPRP compared to sPRP, indicating higher levels of platelet activation in the novel procedure (Figure 4).

### 3.4. Growth Factor Profile in sPRP and nPRP

**Figure 5:**
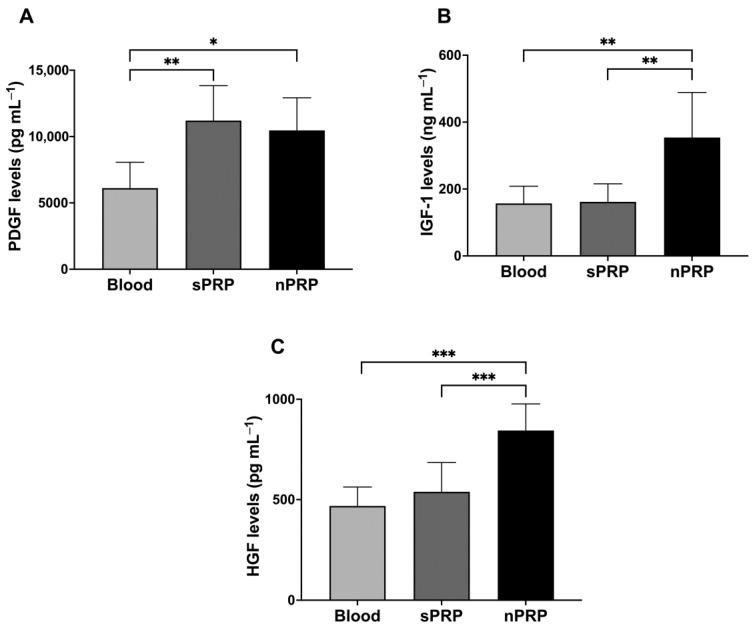
Growth factor levels in blood, sPRP, and nPRP. (**A**) PDGF, (**B**) IGF-1, and (**C**) PDGF-AB growth factor levels are expressed as mean ± standard deviation of 8 donors. Statistical significance was calculated by one-way ANOVA (* *p* < 0.05, ** *p* < 0.01, *** *p* < 0.001).

In order to analyze the levels of platelet and extraplatelet GFs in the sPRP and nPRP, enzyme-linked immunoabsorbent assays (ELISAs) were performed in the platelet lysates of eight donors (Figure 5). A platelet GF, PDGF-AB, and two extraplatelet ones, HGF and IGF-1, were analyzed. Results showed that both sPRP and nPRP doubled PDGF levels compared to blood (*p* < 0.01 and *p* < 0.05, respectively). However, only nPRP showed enrichment of IGF-1 compared to blood and sPRP (*p* < 0.01 in both cases). The same trend was observed in the case of HGF, increasing its levels in nPRP compared to blood and sPRP (*p* < 0.001 in both cases).

### 3.5. In Vitro Analysis of the Bioactivity of the nPRP

**Figure 6:**
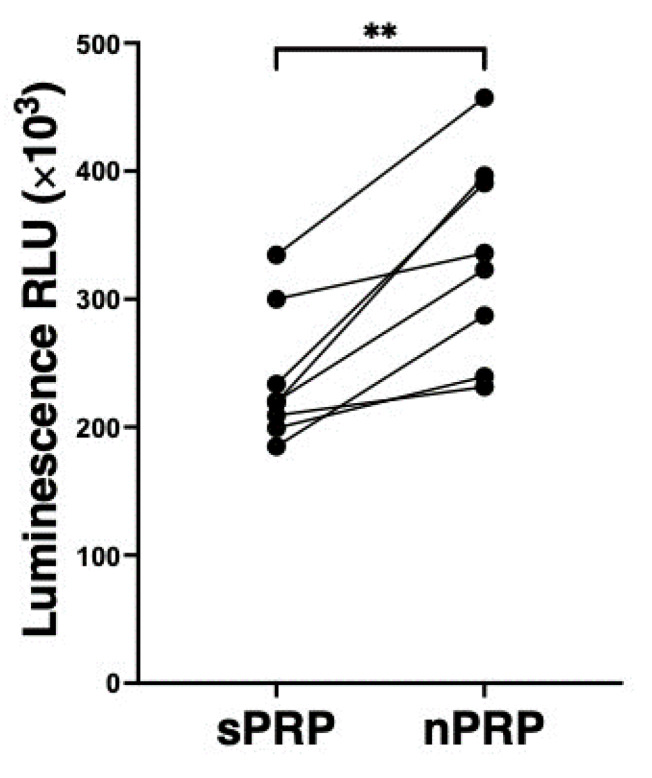
NHDF cell proliferation analysis. The viability levels of the cells incubated with sPRP and nPRP are expressed as relative light units (RLU), and each point represents a different donor (*n* = 8). Statistical analysis was calculated by *t*-test (** *p* < 0.01).

The analysis of the bioactivity was performed by a luminescence-based method, measuring the Relative Light Units (RLU) in NHDF cells cultured with either sPRP or nPRP of 8 different donors. NHDF cultures showed statistically significant higher viability levels at 96 h when treated with nPRP, compared to sPRP (*p* < 0.01) (Figure 6).

## 4. Discussion

While it is well known that extraplatelet factors have regenerative properties, most of the existing methods to produce PRP focus on obtaining plasma enriched in platelets. Even if several processing techniques have been developed to obtain a PRP, none of these methods have the ability to concentrate extraplatelet molecules [23,24]. In the present work, by ultrafiltration and removing water from the plasma, a PRP was obtained where platelets, as well as plasmatic biomolecules present in the plasma, became concentrated. Thus, after activation of the PRP by one of the different existing methods, the platelet content was released, achieving a balance in the concentration factor between platelet and extraplatelet molecules.

Additionally, the ions in the medium remained at the same levels in the nPRP after filtration. This is important since an increase in the concentration of ions is directly related to a change in pH, affecting protein function and, thus, the clotting mechanism [25]. Concerning platelet activation, there was a significant difference in the expression of P-selectin protein comparing both formulas, indicating that in the new methodology, more platelets were activated during the process. This is an expected result since the nPRP method requires several preparation steps with more manipulation. In fact, the step where the protein concentrate is dissolved with the PRP could be key as the concentrate has to be well dissolved, and, therefore, the platelets could be subjected to higher forces and, possibly, be activated. In general, a premature activation of platelets during the PRP-obtaining process could lead to the release of GFs into the medium, potentially causing their loss of functionality. Apparently, this could seem a disadvantage compared to other methods. However, these types of treatments are designed to process the plasma and infiltrate it immediately, so it should not necessarily be a problem since it would not allow for GF degradation in such a short time.

Regarding the analysis of GFs, although platelet-derived GFs depend on the number of platelets, age, and gender influence the concentration of each of the platelet and extraplatelet factors [26], leading to high inter-patient variability. Concerning platelet-derived GFs, since the PDGF depends mainly on the number of platelets [27,28], and as sPRP and nPRP share the property of having doubled the number of platelets compared to basal levels, both PRP contain similar platelet factor concentrations. In fact, in order to obtain nPRP, the protein concentrate is obtained from plasma without platelets with minimal PDGF levels, as reported in a previous study [8], which would not produce a summation effect after dissolving the protein concentrate stuck in the filter with the PRP. Regarding the extraplatelet factors IGF-1 and HGF, it was found that both were concentrated in the nPRP in contrast to sPRP. This confirms that the protein concentrate remaining on the filter that was dissolved in the PRP fraction increased the levels of these factors in nPRP. The benefits of having a higher concentration of IGF-1 could result in a higher stimulation capacity of collagen production, bone formation, keratinocyte migration, and the promotion of cell proliferation [29,30,31]. Moreover, this could be beneficial for elderly patients since it is well known that IGF-1 decreases with age [32]. In line with this, the effect of sPRP and nPRP on the proliferation of NHDF culture was compared. The results showed a higher proliferation capacity in those cells exposed to nPRP. This proliferation response may be promoted by the IGF-1 [8,31,33]. HGF is known to protect tissue from inflammatory damage, as well as to inhibit transcription and translation of pro-inflammatory genes and proteins, resulting in the reduction of pain and inflammation [9,10]. However, no inflammation studies were performed in this work, which opens up a new line of study to further investigate the benefits of this nPRP. Considering the benefits of both extraplatelet GFs, they could potentially make the PRP treatment more effective.

These results make nPRP a novel product as it is able to concentrate molecules that other methods cannot, as is the case of IGF-1 (MW 7.6 KDa) [18]. Furthermore, the novel composition of the nPRP has shown to possess greater bioactivity on cells than sPRP, presenting itself as a potential candidate for future applications in regenerative medicine as it could be in processes involving wound healing. However, other cellular processes, such as cell migration with a scratch assay, reactive oxygen species removal, or anti-inflammation capacity, among others, should be considered to further investigate the properties of this new plasma. It will also be necessary to verify that the higher biomolecule concentration on the nPRP is able to generate real clinical improvements in patients.

## 5. Conclusions

This work shows that the proposed method of ultrafiltration of water from plasma results in a novel PRP, concentrating platelet and extraplatelet GFs, such as PDGF, HGF, and IGF-1. This has an advantage over other systems, which focus on platelet concentration only. With this composition in the nPRP formula, higher induction of proliferative capacity of NHDF cells is achieved compared to sPRP.

Further testing and clinical studies are needed to confirm that this nPRP has therapeutic effects in humans. However, these results open the way to new approaches for the use of nPRP, which are believed to potentially change the course of research for this type of biologic therapy, resulting in products capable of providing more effective treatments to a wider range of patients.

## Data Availability

No new data were created.
